# Histone deacetylase inhibitors induce medulloblastoma cell death independent of HDACs recruited in REST repression complexes

**DOI:** 10.1002/mgg3.1429

**Published:** 2020-07-27

**Authors:** Abdulelah S. Alshawli, Heiko Wurdak, Ian C. Wood, John E. Ladbury

**Affiliations:** ^1^ School of Biomedical Sciences Faculty of Biological Sciences University of Leeds Leeds UK; ^2^ Leeds Institute of Cancer and Pathology University of Leeds St James's University Hospital Leeds UK; ^3^ School of Molecular and Cellular Biology Faculty of Biological Sciences University of Leeds Leeds UK

**Keywords:** CRISPR/Cas9, HDAC, HDACi, medulloblastoma, REST, shRNA

## Abstract

**Background:**

Repressor element 1‐silencing transcription factor (REST) acts as a transcriptional repressor by recruiting several chromatin modifiers, including histone deacetylase (HDAC). Elevated REST expression in medulloblastoma has been associated with tumor progression nevertheless, the tumor shows high sensitivity to HDAC inhibitors (HDACi). However, the functional implications of REST and its requirement for HDACi‐induced anti‐cancer effects are not well understood.

**Methods:**

In this study, the expression of *REST* was evaluated across the medulloblastoma subgroups and subtypes using published gene expression data. Further, the expression of *REST* was modulated using the CRISPR/Cas9 knockout and shRNA knockdown in the Daoy medulloblastoma cell line.

**Results:**

The results of this study showed that the expression of *REST* is elevated in most medulloblastoma subgroups compared to the non‐cancerous cerebellum. Blocking of *REST* expression resulted in increasing the expression of REST‐regulated genes, a moderate decrease in the fraction of the cells in the S‐phase, and reducing the cells' migration ability. However, REST deficiency did not lead to a marked decrease in the Daoy cell viability and sensitivity to HDACi.

**Conclusion:**

The findings of this study indicate that REST is not essential for sustaining the proliferation/viability of the Daoy cells. It also revealed that the anti‐proliferative effect of HDACi is independent of REST expression.

## Graphical abstract

This study investigated the role of REST (DNA binding protein) in the anti‐cancer effects of histone deacetylase inhibitors (HDACi) in a medulloblastoma cell line. The expression of REST was modulated using a CRISPR/Cas9‐based knockout and a small hairpin (sh)RNA knockdown approach in human medulloblastoma Daoy cell lines. It was found that REST expression was not critically required for Daoy proliferation or HDACi‐induced anti‐proliferation effects, suggesting that the HDACi anti‐cancer mechanism is independent of REST expression in the Daoy cells.

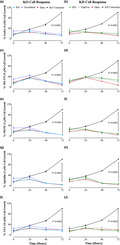

## 1 INTRODUCTION

Medulloblastoma is a diverse group of cerebellar tumors initiated from poorly differentiated neuroectodermal stem cells and characterized by the involvement of the genetic and epigenetic alterations in tumor development and treatment response (Aguilera et al., 2009; Hooper, Hawes, Kees, Gottardo, & Dallas, 2014). Four medulloblastoma subgroups that vary in their clinical outcome, age, gender, and molecular signatures have been identified (Hovestadt et al., 2019). These include; the WNT subgroup, comprising 10% of cases and characterized by the activation of the wingless‐type signalling pathway (Jones et al., 2012); the sonic hedgehog (SHH) subgroup, which represents 20% of medulloblastomas and displays an activated SHH signalling pathway (Pugh et al., 2012); ‘Group 3’ which represents around 19% of cases and occurs exclusively in children, and ‘Group 4’ which makes up to 40% of cases and often exhibits isochromosome 17 amplification (Hovestadt et al., 2019). Recently, gene expression and DNA‐methylation profiling of medulloblastoma tumors revealed a significant heterogeneity within these subgroups, which resulted in further stratification of the medulloblastoma tumors into 12 different subtypes (known as WNTα and β, SHHα, β, γ and δ, Group 3α, β and γ, and Group 4α, β and γ) (Hovestadt et al., 2019). These subtypes are characterized by the activation of different molecular pathways and display different clinical outcomes ranging from favorable to poor prognosis (Cavalli et al., 2017).

Approximately 80% of medulloblastoma tissue samples have been reported to display an elevated repressor element 1‐silencing transcription factor (REST) expression which is concomitant with poor cellular differentiation (Fuller et al., 2005). Medulloblastoma cases with elevated REST expression are often associated with a more dismal prognosis, frequent occurrence of metastasis, and poor overall survival compared to ‘REST‐low’ and ‘REST‐negative’ tumors (Su, Kameoka, Lentz, & Majumder, 2004; Taylor et al., 2012; Wu et al., 2012). Accordingly, REST has been suggested to play a central role in sustaining the tumorigenic potential of medulloblastoma cells due to its ability to block the differentiation of neuronal stem cells which promotes the self‐renewal capacity of tumor cells (Jørgensen et al., 2009; Majumader, 2006; Negrini, Prada, D’Alessandro, & Meldolesi, 2013). REST is a DNA binding protein with nine zinc‐fingers (eight in the DNA binding part and one in the carboxy‐terminal domain [C‐terminal]). The transcriptional repression of REST is induced when it binds to a highly conserved DNA sequence known as repressor element 1 (RE1), which presents in the promoter region of a large number of genes. When REST binds to an RE1 site both its N‐terminal and C‐terminal domains work as hubs to recruit various chromatin remodeling and histone‐modifying enzymes. These include SIN3 transcription regulator family member A (SIN3A), REST corepressor 1 (RCOR1), SWI/SNF‐related, matrix‐associated actin‐dependent regulator of chromatin subfamily a member 4 (SMARCA4), HDAC1, HDAC2, HDAC4, HDAC5, lysine demethylase 1A (KDM1A), and synaptonemal complex protein 1 (SYCP1) (Barrios et al., 2014; Ooi & Wood, 2008).

Histone deacetylases (HDACs) are a group of epigenetic molecular switches that induce their function by binding to several repression complexes including REST. Upon binding to REST, they remove the acetyl group from acetylated lysine amino acid on histone tails which lead to an increase in the electrostatic binding between the histone tails and surrounding DNA, which in turn blocks the access of the transcriptional machinery (Chueh, Tse, Tӧgel, & Mariadason, 2015).

In clinical trials, several HDAC inhibitors (HDACi) have shown promising anti‐cancer activity in treating several hematological malignancies and solid tumors (Eckschlager, Plch, Stiborova, & Hrabeta, 2017). HDACi are a family of anti‐cancer drugs that change the acetylation status of histone and non‐histone proteins causing changes in gene expression, cell metabolic activity, and cell cycle which eventually result in tumor cell death (Häcker et al., 2011). Interestingly, transformed cells are more sensitive to HDACi than normal cells (Eckschlager et al., 2017; Li & Zhu, 2014). However, some tumors exhibit limited sensitivity to HDACi, which could be due to the upregulation of genes that promote tumor growth such as nuclear factor kappa B (*NF*‐*kB*) and anti‐apoptotic protein such as myeloid Cell Leukemia Sequence 1 (*Mcl*‐*1*) (Ma, Ezzeldin, & Diasio, 2009).

In this study, we sought to investigate the function of REST in the Daoy medulloblastoma cell line in order to determine whether REST repression is required for HDACi anti‐cancer effects. The Daoy cell line is a widely used medulloblastoma cell line that was established from a posterior fossa tumor classified as the SHH subgroup and the elevated REST expression in this cell line has been reported in several studies (Fuller et al., 2005; Ivanov, Coyle, Walker, & Grabowska, 2016; Lawinger et al., 2000; Othman et al., 2014; Taylor et al., 2012). To examine REST deficiency, we used a CRISPR/Cas9‐based knockout and a small hairpin (sh)RNA knockdown approach. Our results showed that REST expression was neither critically required for Daoy proliferation, nor for HDACi‐induced anti‐proliferation effects, hence suggesting that the HDACi‐anticancer mechanism is independent of REST expression in the Daoy cells.

## 2 MATERIALS AND METHODS

### 2.1 Ethical compliance

All procedures performed in this study were in accordance with the ethical standards of the University of Leeds.

### 2.2 Cell culture

The human medulloblastoma Daoy cell (ATCC HTB‐186) was cultured in Dulbecco's Modified Eagle's Medium (DMEM, Life Technologies) supplemented with 10% (v/v) Fetal Bovine Serum (FBS, Gibco), and 1% penicillin and streptomycin (Sigma). The cells were cultured at 37°C incubator in 5% CO_2_ and harvested using trypsin‐EDTA (Sigma). All experiments were conducted on cells with fewer than 15 passages.

### 2.3 HDAC inhibitors

SAHA (10009929), MS‐275 (13284), MI192 (18288), and Apicidin (10575) were purchased from Cayman Chemical and dissolved in DMSO (Dimethyl sulfoxide, Sigma). Valproic acid (VPA) was purchased from Sigma (P4543) and prepared in water. The cells were treated with SAHA (5 µM), MS‐275 (5 µM), MI192 (3 µM), Apicidin (3 µM), and VPA (10 mM).

### 2.4 *REST* knockout via CRISPR/Cas9

A CRISPR/Cas9 guide RNA system (OriGene, KN314675) was used to target two different DNA sequences in exon 2 of the *REST* gene (NG_029447.1). Two guide sequences (gRNA1: 5′‐CGCACCTCAGCTTATTATG C‐3′ and gRNA2: 5′‐TGGCAAATGTGGCCTTAACT‐3′) were cloned into the pCas‐Guide vector (which includes a Cas9 expression cassette) by OriGene. The constructs pCas‐Guide1 (OriGene, KN211570G1), pCas‐Guide2 (OriGene, KN211570G2), and the pCas‐Scramble negative control (OriGene, GE100003) were sequenced to confirm the correct insertion sequence. The transfection reactions were performed in 6‐well plates (Nunc) seeded with 3.5 × 10^4^ Daoy cells and cultured in DMEM supplemented with 10% FBS. After 24 h (50–70% confluence), the cells were transfected with one part of pCas‐Guide1 plasmids (2 µg) suspended in Opti‐MEM (Gibco) and three parts (6 µl) of Lipofectamine 2000 transfection reagent (Invitrogen). After 4 h, the transfection media was replaced with 2 ml of complete DMEM and the cultures were re‐incubated for 48 h. The cells were then plated in a 96‐well plate at a cell dilution of 1 cell/100 µl in order to establish monoclonal cell clones. The monoclonal expansion was evaluated microscopically and screening for the genome editing was performed (cells passage 5) by PCR‐amplifying the CRISPR/Cas9 editing site. The products were then sequenced using the DNA Sanger sequencing and the results were compared to the reference human genome. The cells that showed genome editing were propagated for further analysis and the efficiency of *REST* knockout was verified by Western blot analysis using anti‐REST antibody (1:1000, OriGene, TA330562).

### 2.5 Knockdown *REST* expression using shRNA

Two shRNA sequences were designed (shRNA1: 5′‐AGCTAAAAACAGTGTAATCTACAGTAT CACTTCTCTTGAAAGTGGATACTGTAGATTACACT‐3′ and shRNA2: 5′‐GCTAAAAAAGCAGA ATCTGAAGAACAGTTTCTCTTGAAAACTGTTCTTCAGATTCTGCT‐3′) and cloned into pSUPER‐Puro plasmid (Addgene). The constructs were sequenced to confirm their correct insertion and the transfection was performed using Lipofectamine 2000 with 750 ng of the plasmid. After 48 h, the transfected cells were treated with puromycin (Sigma) at a concentration of 5 µg/ml until all the non‐transfected cells had been killed (10 days). The viable cells from the shRNA1 and two transfections were then plated in 96‐well plates (Nunc) at a cell count of 1 cell/100 µl in order to generate monoclonal cell clones. The monoclonal growth was evaluated microscopically and the screening for the pSUPER genome integration was performed using polymerase chain reactions (PCR). The monoclonal cells with low REST expression were expanded and propagated for further analysis.

### 2.6 Cell proliferation and sensitivity to HDACi

The cells were seeded in 96‐well plates at a cell density of 3.5 × 10^4^ cells/ml and cultured at 37°C in 5% CO_2_. The sensitivity to HDACi was measured by replacing the culture media with complete media containing the required concentration of the inhibitors. The growth and sensitivity to the HDACi were assayed after 24, 48, and 72 h using MTT (3‐(4,5‐Dimethylthiazol‐2‐yl)‐2,5‐Diphenyltetrazolium Bromide) assay. The MTT analyses were performed by replacing the culture media with 50 µl of 0.5 mg/ml of MTT reagent (Sigma) prepared in complete culture media and the reaction was incubated at 37°C. After 90 min, the MTT solution was replaced with acidified isopropanol (0.04 M HCL) (Sigma), and the MTT absorbance was measured at 590 nm with a reference filter of 720 nm using a spectrophotometer plate reader (FLUOstar Omega, BMG Labtech).

### 2.7 Wound healing assay

The cells were cultured in 6‐well plates (Nunc) using complete DMEM culture media at a cell density of 1 × 10^5^ cell/ml and incubated at 37°C in 5% CO_2_. After 24 h the growth was examined under an inverted microscope and the cell monolayer (at 90–100% confluence) was scratched by passing a sterile 200 μl pipette tip across the middle of the well in vertical and horizontal directions. The well was then gently washed twice with phosphate‐buffered saline (PBS, Oxoid), fed with serum‐free media, and imaged at the intersection between the vertical and horizontal scratches. A second image was taken 24 h after ‘wounding’ and the cell wound closure was analyzed using the TScratch software (CSElab, Zurich, Switzerland) (Gebäck, Schulz, Koumoutsakos, & Detmar, 2009).

### 2.8 Cell cycle analysis

The cell cycle analysis was performed by harvesting the cells at 80–90% confluence (single‐point analysis) and at 24, 48, and 72 h (time courses analysis). The cells were then suspended in 70% ethanol and stored at −20°C for at least 24 h. Subsequently, the cells were treated with Ribonuclease A (Sigma), stained with Propidium iodide (PI, Sigma), and analyzed on BD LSR II Fortessa flow cytometer using 562–588 nm band pass filter. The percentages of the cells were estimated by deconvoluting the histograms using ModFit LT DNA analysis software (Verity Software House version 3.2).

### 2.9 Western blot analysis

The cells were harvested, resuspended in PBS, 0.5% Triton X 100 (v/v) (Sigma), 0.02% (w/v) sodium azide (Sigma), and 2 mM phenylmethylsulfonyl fluoride (PMSF, Cell Signal) and incubated for 10 min on ice. The nuclei were then pelleted by centrifugation at 4°C and resuspended in 20 mM HEPES‐KOH (Sigma), 420 mM NaCl (Sigma), 25% (v/v) glycerol (Sigma), 1.5 mM MgCl_2_ (Sigma), 0.2 mM EDTA (Sigma), 0.5 mM dithiothreitol (Sigma), 0.2 mM PMSF, supplemented with protease inhibitor cocktail (Sigma) for 20 min on ice. The lysate was centrifuged at 4°C and the supernatant was used in all Western blot analyses.

Western blot was performed on 8% SDS‐polyacrylamide (Bio‐Rad) resolving gels using 80 µg of protein per lane and the separated protein was transferred to a hydrated HYBOND–P PVDF membrane (Amersham). Immunoblotting with primary antibody was carried out at 4°C overnight using a 1:1000 dilution of rabbit anti‐REST (OriGene, TA330562) and a 1:10000 dilution of mouse anti‐β‐actin (Sigma, A1978) antibodies. The detection with secondary HRP‐conjugated antibodies was performed using a dilution of 1:20000 anti‐rabbit (Cell Signalling) and 1:40000 dilution of anti‐mouse (Cell Signalling) antibodies for 1 h at room temperature. Immunoreactive bands were visualized by ECL kit (Enhanced Chemiluminescence, GE Healthcare) and scanned using the LAS‐3000 intelligent dark box (Fujifilm).

### 2.10 Quantitative SYBR green real‐time PCR (qRT‐PCR)

The total RNA was isolated using TRI‐reagent (Sigma‐Aldrich) and reverse‐transcribed using Promega cDNA synthesis reagents. The qRT‐PCR reactions were prepared using 2X SensiMix SYBR & Fluorescein kit (Bioline) with 0.6 µM of forward (FWD) and reverse (REV) primers (*REST* [FWD: 5′‐ACTTTGTCCTTACTCAAGTTCTCAG‐3′, REV: 5′‐ATGGCGGGTTACTTCATGTT‐3′], *RNU6* [FWD: 5′‐CTCGCTTCGGCAGCACA‐3′, REV: 5′‐AACGCTTCACGAATTTGCGT‐3′], *SNAP25* [FWD: 5′‐CGTCGTATGCTGCAACTGGTTG‐3′, REV: 5′‐GGTTCATGCCTTCTTCGACACG‐3′], *SYN1* [FWD: 5′‐AGATTTTTGGGGGACTGGAC‐3′, REV: 5′‐TGTCTTCATCCTGGTGGTCA‐3′], *STMN2* [FWD: 5′‐TGAAGTCGTTTCTCCCCAAC‐ 3′, REV: 5′‐TCACAGCTTGCTCACAATGA‐3′], *GRIA2* [FWD: 5′‐TTGACTTCTCAAAGCCCTTCA‐3′, REV: 5′‐GGCTAAAGGATCAAGAAAGGAA‐3′], *REST* Bi‐allelic genome editing [FWD: 5′‐GCAACATTGGAATGGCCCTG‐3′, REV: 5′‐ATGGCGGGTTACTTCATGTT‐3′]). The amplification was performed on Rotor‐Gene 6000 (Corbett series) using the following PCR conditions: 95°C for 10 minutes, 40 cycles of; 95°C for 10 s, 60°C for 15 s, and 72°C for 20 s. The reaction was then followed by a melting temperature step ramping from 72°C to 95°C. The qRT‐PCR data were analyzed using Rotor‐Gene 6000 Series Software (Qiagen) and the relative gene expression was normalized to the expression of U6 Small Nuclear 1 (RNU6, also known as RNU6‐1) housekeeping gene due to its consistent high expression in transformed cells compared to healthy controls and it is also characterized by its narrow standard deviation (Ma et al., 2018; Manterola et al., 2014; Rice, Roberts, Rai, & Galandiuk, 2015).

### 2.11 Analysis of GEO‐NCBI gene expression data sets

To evaluate the expression of *REST*, *SYN1*, *SNAP25*, *STMN2*, and *GRIA2* in human medulloblastoma and normal cerebellum, we used the Gene Expression Omnibus (GEO, NCBI) data sets; GSE85217 (Cavalli et al., 2017), GSE109403 (Rivero‐Hinojosa et al., 2018), GSE13162 (Chen‐Plotkin et al., 2008), GSE42658 (Henriquez et al., 2013), GSE68776 (Svoboda et al., 2014), and GSE86574 (Amani et al., 2017) (was used for normal human cerebellum), and GSE34101 (Ngo et al., 2014), GSE20492 (Vogel et al., 2010), GSE77947 (Gonçalves da Silva et al., 2017) (was used for the Daoy cell gene expression) (Barrett et al., 2012). The data were analyzed using ExAtlas software (https://lgsun.irp.nia.nih.gov/exatlas/) (Sharov, Schlessinger, & Ko, 2015). The analysis was performed by transforming the values into log2 and the correlation between the data of the selected samples was performed by normalizing the values as described previously (Sharov et al., 2015). The quality of the normalized samples was assessed by the standard deviation and ANOVA with error variance adjustment (also, known as empirical error variance) (Sharov et al., 2015).

### 2.12 Statistical analysis

The significant difference between the means was evaluated using Student's *t*‐test (Unpaired, two‐tailed, assuming equal standard deviation) and One‐Way ANOVA (assuming Gaussian distribution) and *p* value of <0.05 was considered significant. The data were presented as means ± standard error of mean (SEM). Statistical analysis was performed using GraphPad Prism version 8.00 for Windows, GraphPad Software, La Jolla California USA (www.graphpad.com).

## 3 RESULTS

### 3.1 REST expression is elevated in medulloblastoma tumors compared to normal cerebellum

Elevated REST expression in medulloblastoma tumors has been reported in several studies; however, it is unclear whether all medulloblastoma subgroups and subtypes display an elevated REST expression (Fuller et al., 2005; Su et al., 2004; Taylor et al., 2012). In this study, we used the GEO‐NCBI gene profiling data of tissue samples from medulloblastoma patients to explore the difference in the expression of *REST* between the medulloblastoma subgroups. Moreover, we used the gene expression of normal cerebellum data to compare the difference between the subgroups and normal cerebellum. The analysis was carried out using the GSE85217 data set which contains 763 primary medulloblastoma tissue samples. They were classified into the four medulloblastoma (WNT, SHH, Group 3, and Group 4) subgroups and the 12 subtypes based on their genetic and methylation profiles (Hovestadt et al., 2019).

**Figure 1:**
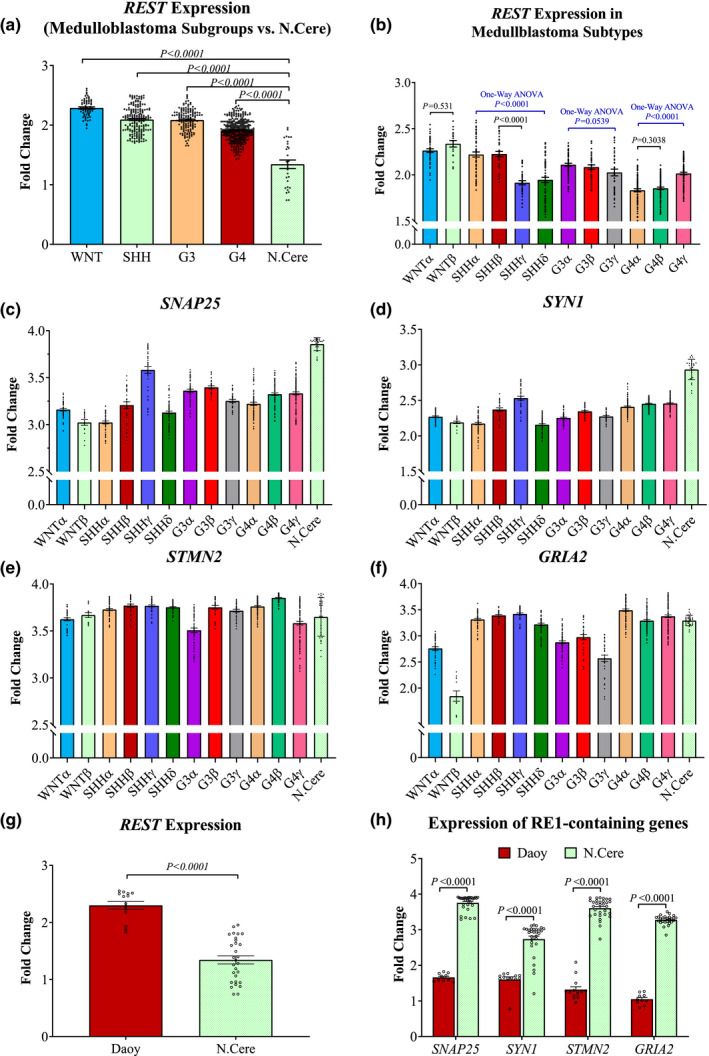
Comparing *REST* expression and its functional effect between medulloblastoma and normal cerebellum tissue samples. The expression results of *REST* (a and b), *SNAP25* (c), *SYN1* (d), *STMN2* (e), and *GRIA2* (f) were extracted from the GEO NCBI database and analyzed using the ExAtlas tool (Sharov et al., 2015). The samples were classified into the four medulloblastoma subgroups (a) and the twelve subtypes (b‐f). (g) Compares the expression of REST between the Daoy cell and normal cerebellum. (h) Displays the expression of some of the RE1‐containing genes in Daoy cells and normal cerebellum. The expression values are represented by the circular dots and the difference between the means was calculated either by using a two‐tailed Student's *t*‐test (a, b, g, and h) or One‐Way ANOVA test (to compare more than 2 groups, b). Error bars depict standard error of mean (SEM). N.Cere, Normal Cerebellum

Comparison of *REST* expression between the medulloblastoma groups and normal cerebellum showed a significant increase in *REST* expression by ~1.6‐fold (*p* < 0.0001) (Figure 1a). Among the medulloblastoma subgroups, the WNT subgroup showed the highest expression of *REST*, whilst the mean *REST* expression was lowest in Group 4. Both of SHH and Group 3 showed approximately equivalent expressions with some variations between the samples of the SHH subgroup (Figure 1a).

We clustered the samples according to the proposed 12 medulloblastoma subtypes in order to demonstrate the expression differences of *REST* between and within the subtypes (Hovestadt et al., 2019). The analysis revealed that the WNT β subtype displayed the highest *REST* expression; however, the difference in the mean between WNT β and α was statistically insignificant (Figure 1b). The results revealed a statistically significant difference between the means of the SHH subtypes as determined by One‐Way ANOVA (*p* < 0.0001). Both α and β subtypes displayed a significant (*p* < 0.0001) elevated *REST* expression by ~0.5%‐fold change compared to γ and δ subtypes. Group 3 showed comparable expression of *REST* between the subtypes (*p* = 0.0539). Within Group 4, there was a significant difference between the means of both α and β subtypes, and they showed the lowest expression of *REST* compared to the γ subtype (*p* < 0.0001) (Figure 1b).

In order to evaluate the functional effect of elevated *REST* expression, we extracted the expression values of REST‐regulated genes that are characterized by high occupancy of REST to their RE1 sites (Bruce et al., 2004; Calderone et al., 2003). These included Synapsin I (*SYN1*), Synaptosome‐Associated Protein 25 (*SNAP25*), Stathmin 2 (*STMN2*), and glutamate Ionotropic Receptor AMPA Type Subunit 2 (*GRIA2*) (Shimojo & Hersh, 2004). The results showed lower expression of *SYN1* and *SNAP25* in the medulloblastoma subtypes compared to normal cerebellum. They also showed a lower expression in the subtypes that displayed an elevated *REST* expression, such as the WNT subtypes, when compared to the subtypes with low *REST* expression, in particular Group 4 (Figure 1b‐f). Unexpectedly, the expression of *STMN2* and *GRIA2* was comparable in the medulloblastoma subtypes compared to the normal cerebellum, apart from the *GRIA2* expression (*p* < 0.001) in the WNT and Group 3 subtypes (Figure 1e,f).

Further, we studied the differential expression of REST between the Daoy cells and normal cerebellum. The results showed that the expression of *REST* in the Daoy cell was two‐fold greater than in normal cerebellum (*p* < 0.0001, Figure 1g). To evaluate whether the elevated *REST* expression had a significant functional effect on the RE1‐containing genes, we compared the expression of REST‐regulated genes between the Daoy cell line and normal cerebellum. The results showed that the expression of these genes was around twofold lower (*p* < 0.0001) in Daoy cells compared to normal cerebellum (Figure 1h).

### 3.2 REST deficiency resulted in the upregulation of RE1‐containing genes

**Figure 2:**
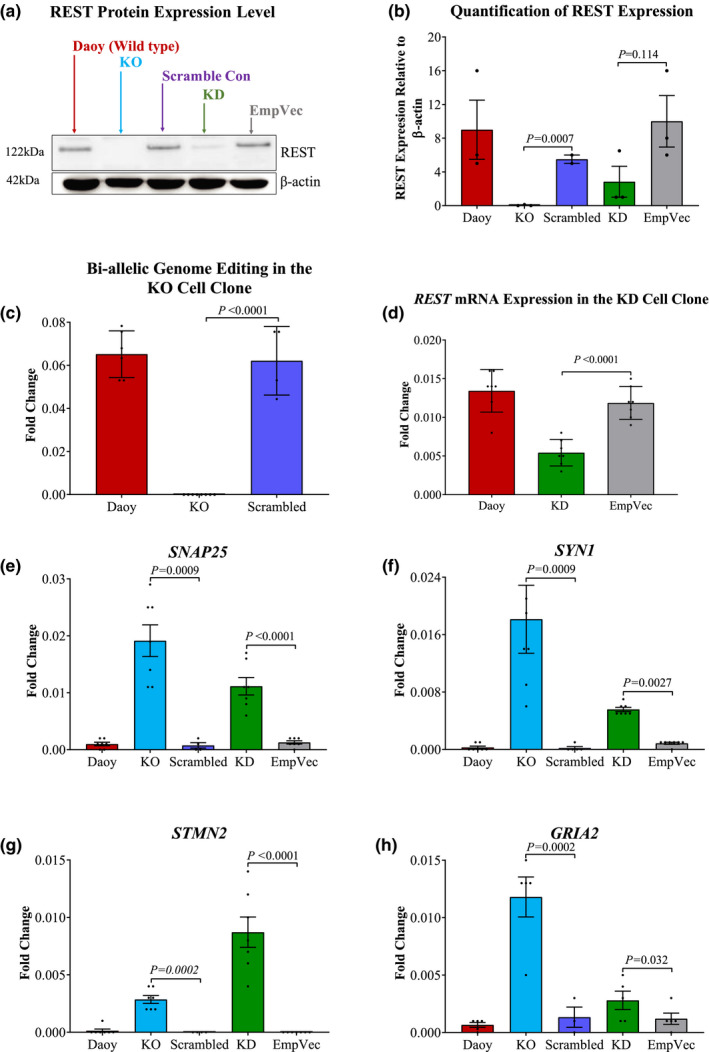
Deficiency of REST expression results in the upregulation of RE1‐containing genes. (a) Western blot analysis of REST expression in the KO and KD cells using anti‐REST polyclonal antibody. (b) The bar chart depicts the densitometric quantification of the signals obtained by Western blotting and the values show the relative REST band intensity relative to β‐actin. The expression of *REST* mRNA in the KO (c) and KD cells (d). The expression of *SNAP25* (e), *SYN1* (f), *STMN2* (g), and *GRIA2* (h) in the KO and KD cells. Data are the averages of 6 biological repeats normalized to the *RNU6* housekeeping gene. The log2 expression values are represented by the circular dots, the *p*‐value was calculated by two‐tailed Student's *t*‐test, and the error bars depict SEM. EmpVec is the shRNA empty vector control

To study the effect of *REST* ‘loss‐of‐function’ on the Daoy cells, and to investigate whether HDACi induce their effect through the recruitment of HDACs into the REST repression complexes, we disrupted *REST* expression using both CRISPR/Cas9 and shRNA approaches. For CRISPR/Cas9‐mediated knockdown, two guide RNA (gRNA) sequences were used to target two different regions of exon 2 of the *REST* gene. For yielding the *REST* knockdown, two shRNA sequences were designed to target exons 2 and 4 of *REST* and selection for stable *REST* downregulation was carried out using puromycin (5 µg/ml for 10 days). The cells afforded by the CRISPR/Cas9 and shRNA approaches were grown at clonal cell densities and the expression of REST was further analyzed by Western blot. A cell clone with a complete absence of REST expression, hereinafter referred to as KO, was identified (Figure 2a). As with most nuclear transcription factor proteins, the abundance of REST expression is low when compared to the highly abundant β‐actin nonetheless, semi‐quantification of protein levels is clearly possible based on these blots (Figure 2b). The biallelic editing of *REST* (exon 2) in the clonally‐derived KO cells was further investigated using a qRT‐PCR reaction with a pair of primers that specifically amplifies the wild‐type sequence and does not give amplification when the target sequence is edited. The results of this analysis showed no amplification with the KO cells which confirm the genome editing of all REST alleles in this clone (Figure 2c).

For the shRNA‐mediated knockdowns, the Western blot analysis identified an expandable cell clone with ~60% reduction in REST expression, hereinafter referred to as KD (Figure 2a,b). The qRT‐PCR results of this clone showed a significant reduction in the *REST* mRNA level by more than 50% compared to the empty vector control (Figure 2d).

In addition, we studied the functional impact of REST deficiency on the expression of the *SYN1*, *SNAP25*, *STMN2*, and *GRIA2* genes between the KO and KD and the control cells. The qRT‐PCR results showed a significant increase in the expression of these genes by more than eightfold in the KO and KD cells (*p* ≤ 0.03), whereas the expression in the KO cell was almost twofold higher than the KD (Figure 2e‐h). Unexpectedly, the expression of *STMN2* in the KD cell clone was approximately onefold higher than its expression in the KO cells (Figure 2g).

### 3.3 REST deficiency does not strongly inhibit cell proliferation but affects the cell migration potential

**Figure 3:**
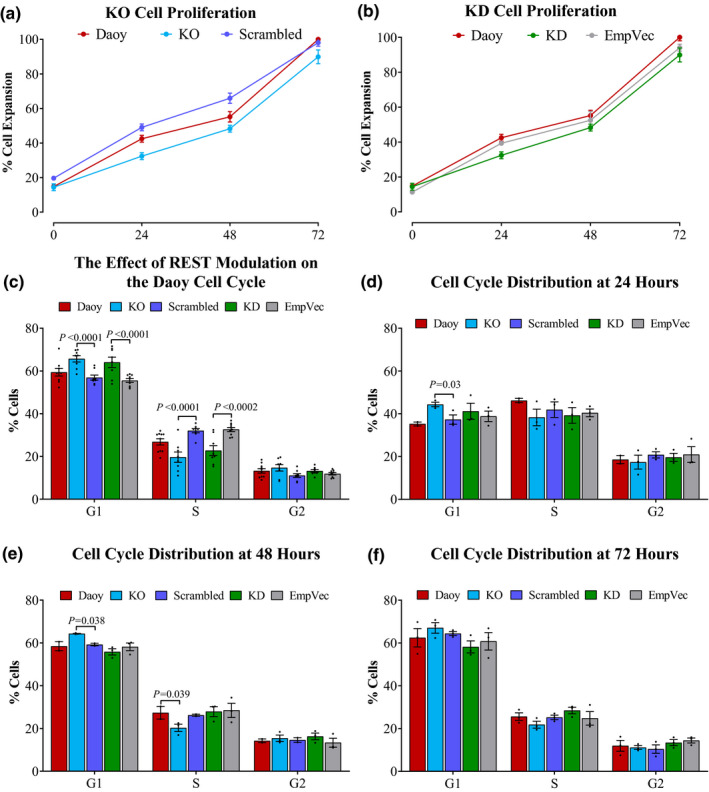
Downregulation of REST expression did not inhibit tumor cell growth and was associated with a marginal delay in cell cycle progression. (a and b) The effect of REST deficiency on Daoy REST KO and KD cell proliferation, respectively. Cellular viability was measured using the MTT assay at 24, 48, and 72 h. The average of three biological repeats is shown as (%) relative to the 72‐h control (the highest reading). Charts (c) displays the effect of REST modulation on the Daoy cell cycle for the 80–100% confluency range. (d) The effect of REST deficiency on the Daoy cell cycle after 24 h (d), (e) at 48 h, and (f) at 72 h from the time point of cell seeding. Data are three biological repeats and the values are depicted as dots, *p*‐value was calculated by two‐tailed Student's *t*‐test and the error bars depict SEM. EmpVec is the shRNA empty vector control

Several studies that modulated REST expression by gene knockdown suggested the direct involvement of REST in reducing cancer cell growth (Conti et al., 2012; Fuller et al., 2005; Kamal et al., 2012; Taylor et al., 2012; Zhang et al., 2016). We examined the effect of REST deficiency on the proliferation of Daoy REST KO and KD cells and on cell cycle using the MTT ‏assay and flow cytometry analysis, respectively. The MTT results revealed only a marginal reduction in the KO and KD cells growth rate compared to REST expressing cells (Figure 3a,b).

The effect of REST deficiency on the cell cycle was evaluated by PI staining and flow cytometry analysis. The KO and KD Daoy cells were harvested at an 80–100% confluency (hereinafter referred to as single time‐point), and at 24, 48, and 72 h from seeding (hereinafter referred to as time‐course analysis). The results of the single time‐point analysis showed an ~5% increase in the accumulation of both the KO and KD cells at the G1 phase (*p* < 0.0001), and a concomitant reduction in the proportion of cells in S phase compared to the control cells (Figure 3c). Consistently, the time‐course analysis showed an increase in the G1 phase accumulation of the KO cells by 5% (*p* = 0.03) at 24‐ and 48‐h before the cell distribution becomes comparable to the control cells at the 72‐h time point (Figure 3e‐f).

**Figure 4:**
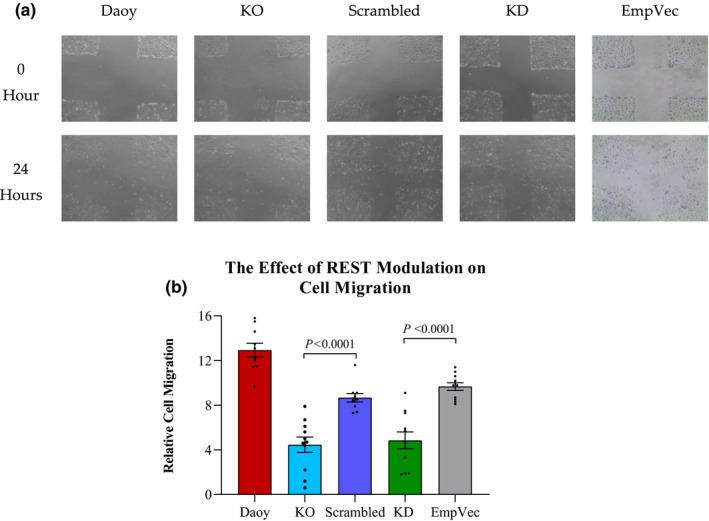
Downregulation of REST expression resulted in a reduced migration ability of the KO and KD cells. (a) In vitro wound scratch assays of Daoy, KO, scrambled, KD, and EmpVec (shRNA empty vector control) cells. (b) The bars show the averages of twelve biological replicates (dots). The *p*‐value was calculated by two‐tailed Student's *t*‐test, and the error bars depict SEM

To assess the effect of REST downregulation on the migration ability of Daoy cells, we used the wound healing assay (Jonkman et al., 2014). The assay was performed by scratching a mono‐cell layer, re‐culturing the cells in a serum‐free medium for 24 h, and the migration ability of the cells was evaluated by their ability to close the scratch. Both the KO and KD cells were ~40% (*p* < 0.0001) less efficient in migration as compared to their control counterparts (Figure 4a,b).

### 3.4 Disrupting REST expression does not reduce the sensitivity of tumor cells to HDACi

**Figure 5:**
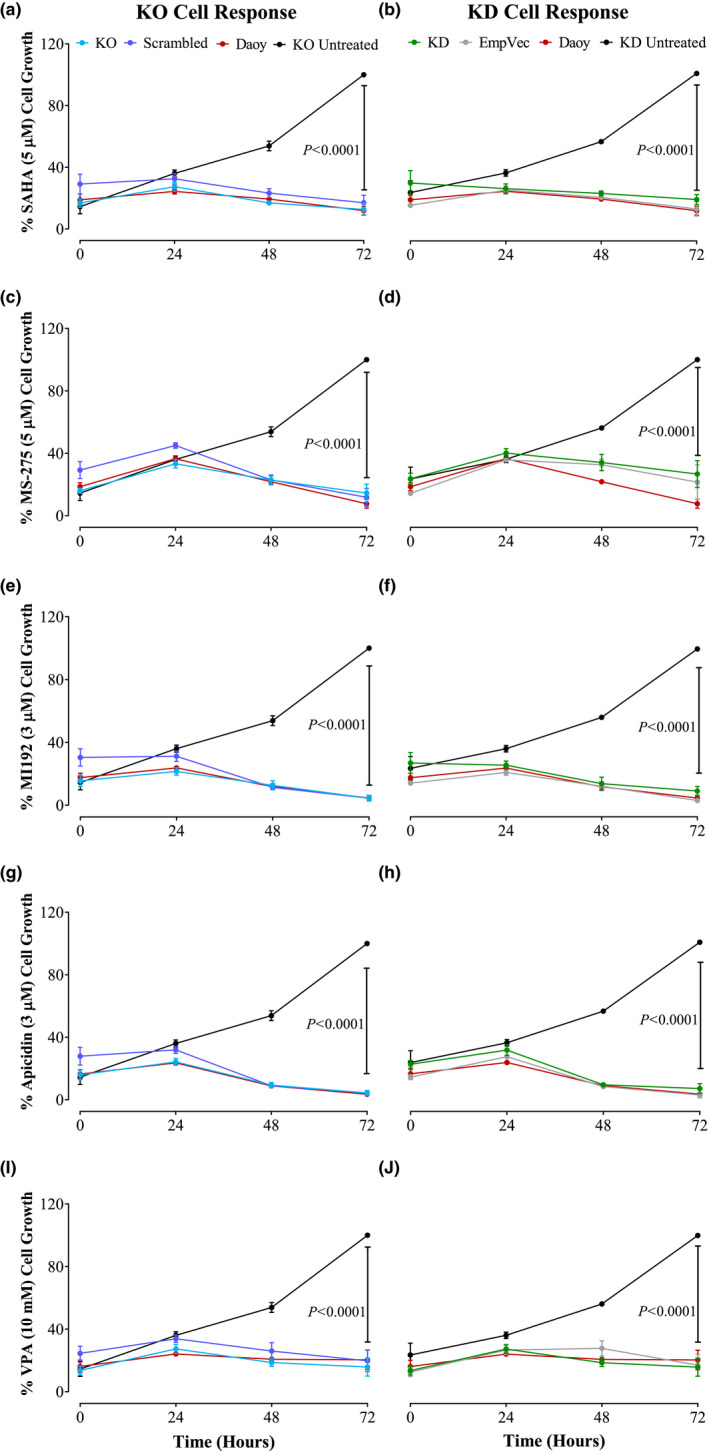
Downregulation of REST expression did not affect the sensitivity of Daoy cells to HDACi. (a and b) The viability of the KO and KD cells treated with a single dose of SAHA, (c and d) MS‐275, (e and f) MI192, (g and h) Apicidin, and (i and j) VPA and was measured with the MTT assay. The measurements were performed at 24, 48, and 72 h from the treatment and the average of three biological repeats is shown as (%) relative to the 72‐h control (the highest reading). The results are the average of three biological repeats, the *p*‐value was calculated by two‐tailed Student's *t*‐test, and the error bars depict SEM. EmpVec is the shRNA empty vector control

In order to examine the sensitivity of REST‐deficient Daoy cells to HDACi, the cells were treated with a single dose of the compounds and cellular viability/proliferation was evaluated over a period of 72 h using the MTT assay. The concentrations of the drugs were chosen to obtain a significant inhibition as described in the literature (Boissinot et al., 2012; Khan et al., 2008; Lauffer et al., 2013). The analysis showed that the rate of cell death in the KO and KD cells was comparable to the one in control cells, which suggests a high sensitivity of the Daoy cell line to all inhibitors used in our study (SAHA (Spiller, Ravanpay, Hahn, & Olson, 2006), MS‐275 (Zwergel et al., 2018), M1192 (Boissinot et al., 2012), Apicidin (Choi et al., 2012), and Valproic acid (VPA) (Li et al., 2005)). The results indicate that the downregulation of REST expression did not significantly reduce the sensitivity of the cells to the inhibitors (*p* < 0.0001) as the Daoy wildtype, KO & KD cells and their control counterparts displayed a similar response to HDACi (Figure 5). This finding may suggest that REST repression complexes are not the primary element of the HDACi‐induced anti‐proliferative effect and that they may act through other HDAC complexes or through non‐histone proteins, which warrants further investigation. It should be noted that this study used one dose of each HDACi for 72 h on the Daoy cell line. Considering that these HDACi are supposed to promote pleiotropic and different effects depending on drug dosage, treatment length, and tumor cell type (Ceccacci & Minucci, 2016).

## 4 DISCUSSION

REST is a transcriptional repressor that plays a significant role in regulating the expression of neuronal genes (Ballas, Grunseich, Lu, Speh, & Mandel, 2005). In several studies, upregulated REST expression in medulloblastomas has been suggested to maintain the self‐renewal potential of tumor cells by repressing the expression of neuronal differentiation genes (Conti et al., 2012; Fuller et al., 2005; Gao et al., 2011; Gonçalves da Silva et al., 2017; Taylor et al., 2012). The transcription repression mechanism of REST is engaged by recruiting several chromatin modifiers such as HDACs into its repression complexes. Both in vivo and in vitro studies demonstrated that treatment with HDACi reduces tumor cell growth; however, it remains unclear whether the high REST expression contributes to the sensitivity of tumor cells due to the domination of HDACs in its repression complexes (Häcker et al., 2011; Ma et al., 2009; Nesterenko, Wanningen, Bagci‐Onder, Anderegg, & Shah, 2012). We sought to identify the contribution of REST in the medulloblastoma cell line (Daoy) proliferation.

In this study, we applied the in silico analysis to explore differences in *REST* expression between the medulloblastoma subgroups using the publicly available gene expression dataset [GSE85217] (Cavalli et al., 2017). The analysis revealed a wide variation in *REST* expression between the medulloblastoma subgroups, in particular, with the SHH subgroup and these findings are in line with the known medulloblastoma heterogeneity (Hovestadt et al., 2019). The expression of the analyzed RE1‐containing REST target genes was overall reduced in medulloblastoma as compared to normal cerebellum tissue specimens. For example, the expression of *SNAP25* and *SYN1* showed a significant reduction in their expression compared to normal cerebellum. Yet, the expression data of *STMN2* and *GRIA2* did not show a significant difference when compared to normal cerebellum tissue specimens.

We established REST loss‐of‐function Daoy cell clones (using CRISPR/Cas9 knockout and shRNA knockdowns) and they showed a sustainable proliferative potential. Comparing the REST‐deficient cells to the wild‐type cell showed a significant increase in the expression of some RE1‐containing genes; however, it did not result in differentiating the tumor stem cell or interrupting the oncogenic regulation. Noteworthy, the number of REST regulated genes has been estimated to exceed 2000 genes (Otto et al., 2007). Despite the enormous effect of REST modulation though, the REST deficient cells maintained their proliferative potential and did not show any morphological changes or cell death. These findings suggest a non‐critical role for REST in regulating tumor cell proliferation and differentiation and may point to the existence of other oncogenic regulations that maintain the self‐renewal potential of tumor stem‐like cells. This conclusion is further supported by the findings of several of microarray and next‐generation sequencing gene expression studies that were performed using primary human medulloblastoma; however, the findings of these studies did not suggest REST as a factor in sustaining the tumor cell growth (Cavalli et al., 2017; Hooper et al., 2014; Jones et al., 2012; Thompson et al., 2006). It should be noted that the conclusion of our study was derived from one experimental cell line which represents the SHH medulloblastoma subtype, hence it is not known whether other cell models would show comparable response to REST deficiency. Additionally, the depletion of REST expression was performed in vitro, which lacks important factors of the tumor microenvironment. In vivo experiments, preferably patient‐derived xenograft mouse models should clarify the relationship between REST depletion and tumorigenesis in medulloblastoma with the context of the microenvironment. Collectively, our results indicate elevated REST expression in the Daoy cells and the functional impact of REST deficiency on the expression of the RE1‐containing gene.

Further, the study has found that blocking the REST expression led to a significant decrease in migration in the KO and KD cells. This finding is further supported by a previous study which revealed that knocking down *REST* in glioblastoma U‐87 and U‐251 cell lines, using shRNA, resulted in inhibiting migration in glioblastoma cells yet, it did not stimulate cell death or reduce tumor size (Zhang et al., 2016).

We also found that the depletion of REST expression resulted in a marginal accumulation of Daoy cells in the G1 phase concomitant with a slight decrease in the S phase. The conclusion of this work aligns with some of the recent research findings. For example, Das et al., (2013) used shRNA to knockdown *REST* expression in the Daoy cells to demonstrate that the loss of REST resulted in increased accumulation in G1 phase with a concomitant decrease in the S phase without leading to cellular apoptosis (Das et al., 2013). They also reported that the loss of REST expression led to a decrease in the expression of MYCN (a proliferative marker) and an increase in the expression of certain antiproliferation markers such as H3 histone pseudogene 23 (H3P23) and USP37 (Das et al., 2013). USP37 is an element of the ubiquitin system and controls the cell cycle by stabilizing cyclin‐dependent kinase inhibitor 1B (CDKN1B) which plays a major part in slowing down the progression through the G1 phase (Das et al., 2013). However, the association of USP37 with REST needs further investigation.

To the best of our knowledge, this is the first study to use a knockout approach to study the contribution of REST in medulloblastoma. Several former studies used knockdown approaches in their analysis and they suggested REST as a key contributor in tumor cell growth and apoptosis and recommended REST as a promising target for medulloblastoma treatment (Fuller et al., 2005; Su et al., 2004, 2006; Taylor et al., 2012). Moreover, previous studies used a non‐functional form of REST (known as REST‐VP16, competes with REST and activates RE1‐containing genes) in a Daoy xenograft mouse model. These studies reported that the expression of the non‐functional form of REST resulted in reduced tumor cell growth via the induction of apoptosis in the in vivo intracranial medulloblastoma (Fuller et al., 2005; Lawinger et al., 2000).

We hypothesized that HDACi induce their effect through REST repression complexes which recruit several HDACs (Barrios et al., 2014; Ooi & Wood, 2008). Our results showed that disrupting REST activity alone did not affect the sensitivity of the cell to the HDACi. This may indicate that these inhibitors do not entirely depend on the REST recruited HDACs and they possibly arrest cancer growth through other HDACs complexes and/or non‐histone protein yet, it requires further investigation. A previous study has suggested REST overexpression as a marker for HDACi treatment; however, the findings of this present study do not fully support this suggestion (Taylor et al., 2012).

Our findings suggest that the Daoy medulloblastoma cells exhibit comparable sensitivity to HDACi despite the variation in the HDAC‐selectivity between the drugs. For example, the HDACi MS‐275 specifically inhibits HDAC1 and to a lesser extent HDAC2 and HDAC3 (Adhikari, Amin, Trivedi, Jha, & Ghosh, 2018), MI192 and Apicidin inhibit HDAC2 and 3 (Boissinot et al., 2012), while SAHA and VPA inhibit class I/II and I/IIα HDACs, respectively (Micelli & Rastelli, 2015). Comparing our results obtained via MS‐275 treatment with other studies that examined other medulloblastoma cell line (D283) suggests the high sensitivity of the Daoy cells to MS‐275 treatment as D283 showed a minor sensitivity to this inhibitor (Aguilera et al., 2009). It is not known why medulloblastoma cells exhibit different sensitivity to HDACi though, we may hypothesize that each medulloblastoma cell line has a different genetic signature that dictates its response to a particular inhibitor. The sensitivity result of SAHA in this study is in agreement with the findings of a previous study that examined Daoy, D283, and D341 medulloblastoma cell lines (Spiller et al., 2006). The ability of SAHA to induce cell death among different medulloblastoma cell lines could be due to the efficiency of SAHA to inhibit a wider range of HDACs which includes all Class I/II HDACs.

To conclude, the findings of this study revealed that the disruption of REST expression in the Daoy experimental model resulted in the de‐repression of neuronal genes, non‐significant/marginal delay in the progression of the cells at the G1 phase of the cell cycle, and a significant reduction in the cell migration ability. However, the downregulation of REST was not sufficient to induce a significant effect on the Daoy proliferation and viability.

## CONFLICTS OF INTEREST

The authors certify that they have no affiliation with or involvement in any organization or entity with any financial interest, or non‐financial interest in the subject matter or materials discussed in this manuscript.

## AUTHOR CONTRIBUTION

All authors have read and approved the published version of the manuscript. Conceptualization, Abdulelah S. Alshawli and Ian C. Wood; methodology, Abdulelah S. Alshawli and Ian C. Wood; designed and performed formal analysis, Abdulelah S. Alshawli; performed the experiments, analyzed data, writing original draft; review and editing, Abdulelah S. Alshawli, Heiko Wurdak, Ian C. Wood, and John E. Ladbury; visualization, Abdulelah S. Alshawli; supervision, John E. Ladbury, Heiko Wurdak, and Ian C. Wood; project administration, John E. Ladbury; funding acquisition, John E. Ladbury.
